# Assessing academic anxiety in d/Deaf, DeafBlind, and hard of hearing individuals

**DOI:** 10.3389/fpsyg.2026.1663756

**Published:** 2026-01-29

**Authors:** Rachel Gabriella Pizzie, Christina Eun-Young Kim, Rachel Marie Sortino, Rachel Inghram

**Affiliations:** 1 Educational Neuroscience Program, Gallaudet University, Washington, DC, United States; 2 Clinical Psychology Program, Gallaudet University, Washington, DC, United States

**Keywords:** academic anxiety, American sign language (ASL), anxiety, Deaf, hard of hearing, math anxiety, science anxiety, writing anxiety

## Abstract

Students encounter many challenges to academic success. Whereas some students thrive in stressful environments, other students falter. Some students also encounter social and emotional factors that might detract from academic achievement, including negative emotions like stress and anxiety. Academic anxieties refer to negativity, nervousness, and avoidance associated with different academic domains, such as math anxiety, science anxiety, test anxiety, trait (general) anxiety, and writing anxiety. Not only do individuals with high academic anxiety experience stress, but high academic anxiety is associated with decreased academic performance in the specific domain. On average, d/Deaf, DeafBlind, and hard of hearing (DDBHH) people show decreased academic performance compared to hearing populations, but more research is needed to understand how academic anxieties may play a role in creating challenges for DDBHH students. In the present study, we explored the reliability of the Academic Anxiety Inventory (AAI) in a DDBHH sample (*N* = 145). In this study, each AAI item was presented in both American Sign Language (ASL) and English, providing additional accessibility to DDBHH participants. Results showed that each of the five subscales of the AAI had high reliability. Moreover, a factor analysis showed each of the hypothesized five subscales of the AAI were represented by corresponding factors in this sample. In this DDBHH sample, the AAI showed relatively low intercorrelation between domains of anxiety, demonstrating that the domains of anxiety are relatively independent and separable from one another. Additional results compared the DDBHH with a sample of hearing people from the original psychometric validation of the AAI and explored other relations between the AAI and language background. Overall, these results suggest that the AAI is a reliable and appropriate questionnaire for use with DDBHH participants. Understanding and addressing academic anxieties in DDBHH communities is a priority for encouraging academic achievement. Developing appropriate, culturally sensitive, and accessible ways to reduce the impact of these anxieties is vital to encourage DDBHH students to achieve their potential.

## Introduction

Whereas some students are able to thrive in stressful environments, some students respond to stress with academic anxiety, or negative, apprehensive, anxious emotions associated with different domains of academic content (Ashcraft, 2002; Kim et al., 2025; Pizzie and Kraemer, 2019). In this study, we explored academic anxieties in a sample of d/Deaf, DeafBlind, and hard of hearing (DDBHH) people (*N* = 145). Here we use the term DDBHH to refer to d/Deaf communities, recognizing the diversity and heterogeneity of these communities and inclusion of DeafDisabled, Deaf Autistic, and Late Deafened individuals, and individuals whose identities are not specifically named here. Some members of DDBHH communities experience additional barriers to educational success created by lack of accessibility in educational environments. This research explored a novel gap in the research literature addressing how academic anxieties might present in DDBHH communities. Our study explored the reliability of the Academic Anxiety Inventory (AAI; Pizzie and Kraemer, 2019) when the questionnaire was presented bilingually in American Sign Language (ASL) and written English, providing additional accessibility for DDBHH individuals who benefit from materials presented in a primary or preferred accessible language. We (1) evaluated the psychometric properties of the questionnaire when administered to a DDBHH sample (2) explored whether DDBHH individuals experienced increased anxiety in specific academic domains, and (3) investigated whether self-reported language background was related to writing anxiety. Our overall goal was to evaluate whether the AAI measure was an appropriate and reliable measure of multiple domains of academic anxiety in DDBHH individuals, opening up future avenues to investigate how academic anxiety might create barriers or challenges for academic success.

Although it is potentially useful to recognize patterns of negative affect and avoidance across academic domains to identify parallels and consistencies in these emotional reactions and outcomes, it seems that many individuals who experience academic anxieties only experience these reactions associated with specific domains (Kim et al., 2025). In this way, academic anxieties may be better conceptualized as academic phobias, where the individual learns and develops specific fearful responses and avoidance toward an academic subject (Cuder et al., 2025; Pizzie and Kraemer, 2017). Perhaps the most well-studied of these domains is mathematics anxiety, wherein individuals develop intense fearful, anxious, and avoidant responses to anticipating and encountering mathematics (Lyons and Beilock, 2012), doing math calculations (Ashcraft and Faust, 1994; Ashcraft and Krause, 2007; Pizzie et al., 2020a; Pizzie et al., 2020b), as well as long-term educational outcomes such as chosen majors and careers (Betz, 1978; Daker et al., 2021, 2023; Hembree, 1990). Other examinations of academic anxiety have also explored anxiety about science (Bryant et al., 2012; Mallow, 2010, 2014), and anxiety about language skills (Cheng et al., 1999; Daly and Miller, 1975; Horwitz, 2016; Horwitz et al., 1986; Ramirez et al., 2018a), including bilingual language skills in DDBHH individuals (Kim et al., 2025).

The Academic Anxiety Inventory (AAI) is a 50-item questionnaire designed to measure academic anxiety associated with mathematics, science, writing, test, and trait anxiety (Pizzie and Kraemer, 2019). The questionnaire is comprised of five 10-item subscales associated with anxiety in each domain, reducing the number of overall questions a participant might have to answer compared to completing multiple separate questionnaires to assess each type of academic anxiety. The AAI measure was originally developed to primarily assess math anxiety, while showing it was distinct from the other domains of academic anxiety.

This prior work addressed necessary theoretical questions about the development of math anxiety and other academic anxieties, investigating whether math anxiety and other specific academic anxieties could be meaningfully differentiated from broader patterns of anxiety, such as test anxiety and trait anxiety (Cipora et al., 2022; Daker et al., 2022; Pizzie and Kraemer, 2019). Results suggested that previous self-reported measures of academic anxiety were not necessarily measuring independent domains of anxiety. For example, a popular measure of math anxiety, the Mathematics Anxiety Rating Scale (Richardson and Suinn, 1972; Suinn and Winston, 2003), was highly related to test anxiety (correlations of *r* = 0.60–0.75; Kazelskis et al., 2000; Pizzie and Kraemer, 2019). However, by selecting more independent questions that better represented each unique domain, the subscales of the AAI reduced the intercorrelation between domains compared to previously published questionnaires, providing evidence that each of the five domains of anxiety could be independently measured. The development of this questionnaire addressed a significant gap in the literature by supporting the specificity of different domains of academic anxiety. In addition, math anxiety measured by the AAI-Math subscale in hearing populations has been associated with measures of mathematics performance in lab tasks, fMRI measures of arithmetic performance, and performance in real-world classrooms (Pizzie et al., 2025; Pizzie et al., 2020a; Pizzie and Kraemer, 2019, 2023).

Anxiety and performance likely have a reciprocally detrimental relationship in which decreased performance leads to more anxiety, and more anxiety leads to decreased performance (Carey et al., 2016; Ma et al., 2004). These kinds of academic anxiety are related to a downward spiral in learning and achievement, where weaknesses in conceptual understanding and anxious feelings are associated with avoidance of the material, leading to less understanding and greater feelings of anxiety associated with academic content (Kim et al., 2025; Ramirez et al., 2018b; Suárez-Pellicioni et al., 2015). Students who may struggle to learn academic concepts may be at greater risk of developing academic anxieties, and as feelings of anxiety begin to compound, these students may continue to underperform and may avoid learning and studying these topics (Choe et al., 2019; Daker et al., 2023; Jenifer et al., 2022). Students who encounter barriers created by a lack of accessibility, such as some students who are DDBHH (Solomon, 2021), may be at increased risk for developing academic anxieties. Lack of accessibility additionally creates challenges to develop knowledge and expertise, potentially leading to a weaker conceptual foundation and more anxiety associated with that topic.

DDBHH individuals are heterogeneous, and individuals in these communities are extremely diverse in their educational and linguistic backgrounds. In terms of scope, in 2008, the American Community Survey estimated that there were approximately 11 million people in the US who reported hearing loss, and 341,288 school-aged children (between the ages of 6 and 18) who had difficulty with hearing loss (Walter, 2010). DDBHH communities are characterized by a wide variety of access to sound and language backgrounds, including varying experience and expertise with American Sign Language (ASL) and English (Garberoglio et al., 2019; Humphries et al., 2014; McKee et al., 2013). DDBHH individuals also have a wide variety of educational experiences, such as “mainstream” school environments (approximately 85% of DDBHH students) where students spend varying amounts of time integrated in classes with hearing peers and may or may not have adequate accommodations such as ASL interpretation, captions, or assistive devices (Walter, 2010). Some deaf students attend specialized schools (approximately 12% of DDBHH students), such as K-12 schools tailored to DDBHH students, some of which provide instruction in ASL (Walter, 2010). In a subsample of students who provided information about their educational environments in 2013–2014, approximately half of DDBHH students received instruction in spoken language only, 20% received instruction in signed language only, and approximately 25% used some form of sign-supported speech such as “SimCom” or cued speech (Affairs, 2014). On average, DDBHH people have decreased academic achievement and attain lower levels of education than hearing peers (Garberoglio et al., 2019). For example, 15% fewer deaf people attain a bachelor’s degree compared to hearing peers. Although trends seem to be improving, with more young DDBHH individuals attaining more years of education, still very few DDBHH individuals attain postsecondary degrees (~7%), and on average, have lower employment rates compared to hearing people across degree fields/specialties (Garberoglio et al., 2019).

DDBHH students may contend with additional challenges to learning content created by a lack of accessibility (Solomon, 2021). Some DDBHH students depend on learning information through an interpreter who may not have expertise in the subject-matter, or may rely on written-English materials, which may not be presented in a preferred language modality, such as ASL. Because of these additional accessibility challenges, some DDBHH students may have a weaker conceptual foundation, and could be predisposed to develop academic anxieties. Moreover, DDBHH individuals may be more likely to be diagnosed with anxiety or depression, and at earlier ages (Kushalnagar et al., 2019). Although general (trait) anxiety is considered to be an independent construct from academic anxieties, it is moderately correlated with some types of academic anxiety (Daker et al., 2022; Hembree, 1990) and experiencing more general anxiety may also predispose people to develop specific anxieties associated with academic domains.

Many DDBHH individuals struggle to gain proficiency in English and ASL, especially DDBHH children who are not given access to a visual signed language from infancy (Finton et al., 2024; Hartman et al., 2019; Humphries et al., 2012; Kim et al., 2025; Kushalnagar et al., 2020b). Because of these challenges to developing proficient language skills, some DDBHH individuals may be predisposed to developing language anxiety associated with ASL and English (Kim et al., 2025). Specifically, we sought to investigate whether DDBHH people would report increased anxiety associated with a specific language skill: writing. In this study, we collected self-reported data related to participants’ language background. Given past research on the benefits of bilingualism and signed language, exploring language anxiety is vitally important to understanding academic barriers for DDBHH students.

As in the scientific literature focusing on the hearing community, most of the academic anxiety research in DDBHH participants has focused on math anxiety, as it is the most well-characterized (Ariapooran, 2017; Mishra et al., 2022; Pizzie et al., 2025). Past research suggests that deaf and hard of hearing (DHH) individuals may experience increased average math anxiety compared to hearing individuals (Ariapooran, 2017; Mishra et al., 2022; Note: these studies do not report any participants who are DeafBlind, so here we focused on self-reported DHH hearing status), or may experience increased science anxiety compared to hearing individuals (Pizzie et al., 2025). However, more nuance is needed in understanding how DHH students experience math anxiety, such that the relations between math anxiety and math attitudes and parental behaviors differed between DHH and hearing students (Mishra et al., 2022). Moreover, this study reported a positive correlation between attitudes toward math and school environment and math anxiety, such that DHH students who had positive feelings toward math and felt that their school environment was supportive also had increased math anxiety. These results were counterintuitive, and the authors suggest that because of the pervasive underrepresentation of DHH students in STEM fields, increased positive relations between math attitudes, school environments and math anxiety reflect an interest and desire to succeed in STEM but a recognition that these aspirations may not come to fruition. DHH students might have goals and interests related to STEM, but fear poor performance and failure due to the vast underrepresentation of DHH individuals in these fields (Mishra et al., 2022).

In another study with DHH adults, math and science anxiety were negatively associated with self-reported interest in studying STEM fields and were inversely associated with performance on a test of visuospatial skills, which have been shown to be positively associated with STEM outcomes (Pizzie et al., 2025). Moreover, when predicting self-reported interest in studying STEM fields, math and science anxiety accounted for more variability than participants’ performance on a visuospatial skills task. These results indicated that anxiety, even more so than cognitive ability, might be more closely associated with interest in pursuing STEM (Daker et al., 2021; Pizzie et al., 2025). For DDBHH and hearing individuals alike, academic anxieties like math anxiety and science anxiety seem to create barriers for learning and performing important academic skills. Measuring and understanding feelings of academic anxiety across various domains in DDBHH individuals is a priority for future research seeking to improve academic achievement in these communities. In order to develop better and more accessible interventions to reduce anxiety and improve academic performance, it is essential to develop tools to assess different types of academic anxiety. In addition to improving accessibility in educational environments, there is room for developing novel innovations and investigating avenues of support for DDBHH students in educational environments to encourage academic achievement by reducing academic anxiety.

In this study, we sought to assess the reliability of the AAI (Pizzie and Kraemer, 2019) in a DDBHH sample. We investigated if this questionnaire would be an appropriate and reliable measure within this DDBHH sample to better understand experiences of academic anxiety. We presented the AAI bilingually, using written English questionnaire instructions which were paired with optional ASL translation videos. The ASL translation videos for the instructions and items were intended to provide additional accessibility for DDBHH individuals who rely on ASL and who would benefit from additional understanding provided by the questionnaire information in a signed language. Our goal was to assess the psychometric properties of the bilingual questionnaire, expecting that measures of reliability, factor analysis, and the independence of each of the subscales would mirror the results from the original psychometric analyses of the AAI. We hypothesized that the AAI subscales would show high reliability, such that we predicted that Cronbach’s alpha for each subscale would be above a criterion of 0.7. We also expected that a confirmatory factor analysis would demonstrate that each questionnaire item would be closely related to a factor representing each of the subscales. Further, we hypothesized that the factor structure would be closely related to the factor structure shown in the original AAI validation sample. In a subset of individuals who took the AAI at least twice, we also predicted that the AAI would have high test–retest reliability, as was demonstrated in the original publication validating the questionnaire.

In addition, we also wanted to explore novel research questions in a DDBHH sample. First, as previously described, we wanted to evaluate whether DDBHH individuals self-reported increased mean anxiety levels across the five subscales. We hypothesized that DDBHH individuals might report increased general (trait) anxiety as measured by the AAI-Trait subscale (Kushalnagar et al., 2019). We also predicted that our sample would report increased math and science anxiety (Ariapooran, 2017; Mishra et al., 2022; Pizzie et al., 2025). We also sought to evaluate the relationships between writing anxiety (writing in English), and self-reported preferences for English and ASL. We predicted that we would see consistency in the attitudes toward English, such that positive preferences toward English as a communication strategy would also be reflected in tandem by reduced writing anxiety. We also predicted that we might observe positive benefits of ASL, such that increased comfort or preferences for using ASL would also have positive bilingual benefits such that they would be related to reduced writing anxiety. This study represented a unique opportunity to begin to explore the relations between language background and language anxiety in a DDBHH sample.

Our goal was to explore the following Research Questions (RQs):

1. In a DDBHH sample, is the AAI a sufficiently reliable measure?
  - Using Cronbach’s alpha, do all subscales have sufficient reliability?
  - Exploring the factor structure of the AAI subscales, are hypothesized domains of math, science, writing, trait, and test anxiety represented in the data?
  - Does the AAI have sufficient test–retest reliability in a DDBHH sample?
2. Do all the subscales represent related but independent factors?
3. Do DDBHH individuals report increased academic anxiety compared to a hearing sample?
4. How does writing anxiety relate to language preferences in a DDBHH sample?

## Methods

### Participants

In this study, all participants (*N* = 145) completed an online version of the AAI (Pizzie and Kraemer, 2019) combined with other surveys or tasks related to academic performance. In order to be eligible for inclusion in this dataset, participants had to report that they were d/Deaf, hard of hearing, DeafBlind, or self-described with an identity that included hearing loss (for example “Deaf Disabled”), and between the ages of 18–65. Participants who reported that they were “hearing” were excluded from the study. Some participants (*n* = 98) completed this questionnaire as part of an online study. There were no inclusion or exclusion criteria based on ASL proficiency to be included in these analyses, but some individual studies had more specific criteria related to ASL skills. Some participants completed this survey as part of a pre-test battery of surveys for an in-lab (*n* = 18) or in-classroom experiment (*n* = 29). Participants in the in-lab study (*n* = 18), had to report advanced knowledge of ASL due to other tasks included in that study. A subset of participants (*n* = 45) participated in more than one of these studies and thus completed the AAI more than once, enabling us to evaluate the test–retest reliability of the AAI subscales. With respect to self-reported Education Level, *n* = 47 individuals had no self-reported level of education. This included participants from the in-classroom experiment (*n* = 29), where all participants were undergraduates and education level was not assessed; for the purposes of that study, all participants would have reported identical levels of education. Because of the recruitment of those participants, it can be assumed these individuals had some undergraduate education. However, this was reported as “*NA”* in the education level demographics because it was not self-reported by the participants. An additional *n* = 18 individuals did not report education level, for a total of *n* = 47 participants who did not have an available education level reported. Self-reported demographics related to self-reported age, gender, race, and Latino/a/x ethnicity are included in Table 1. Descriptive comparisons of AAI subscale scores between the d/Deaf and Hard of Hearing samples are included in Supplementary Figure 1.

**Table 1:** Demographics of DDBHH sample (*N* = 145).

Variable	Mean (SD)	Range
Age (in years)	27.86 (9.55)	18–57
Years of ASL	19.92 (12.19)	0.2–52
Gender	*N*	%
Male	52	35.86%
Female	73	50.34%
Non-binary	14	9.66%
Self-describe	4	2.76%
Prefer not to say	2	1.38%
Race		
White	103	
Black or African American	17	
American Indian or Alaska Native	4	
Asian	15	
Native Hawaiian or Pacific Islander	2	
Self-describe	5	
Prefer not to say	6	
Latinx		
Yes	20	13.79%
No	119	82.07%
Prefer not to say	6	4.14%
Hearing Status		
D/deaf	101	69.66%
Hard of Hearing	39	26.90%
Hearing	0	0.00%
DeafBlind	3	2.07%
Self-describe	2	1.38%
Assistive Device Use		
1 + Cochlear Implant	25	17.24%
1 + Hearing Aid	51	35.17%
Do not use	50	34.48%
Do not need	15	10.34%
Self-describe	4	2.76%
Education Level		
Completed some high school	0	0.00%
Graduated high school	10	6.90%
Completed GED	0	0.00%
Completed 1 year of undergraduate education	9	6.21%
Completed 2 years of undergraduate education	6	4.14%
Completed 3 + years of undergraduate education	18	12.41%
Graduated from a 4-year undergraduate institution with a degree (Bachelor’s degree)	12	8.28%
Graduated from a 2-year undergraduate institution with a degree (Associate’s degree)	3	2.07%
Completed 1 + years of post-secondary education (some graduate education)	11	7.59%
Graduated with a professional degree (JD, MD, DO)	0	0.00%
Graduated with a post-secondary degree (Masters, PhD)	29	20.00%
*NA*	47	32.41%
Age of Acquisition: American Sign Language (ASL)		
Infant (age birth–2 years old)	54	37.24%
Toddler (age 2–4 years old)	15	10.34%
Young Childhood/Elementary School-Aged (age 4–10 years old)	26	17.93%
Pre-teen/Middle School-Aged (age 10–13 years old)	8	5.52%
Adolescent/High School-Aged (14–18 years old)	19	13.10%
Young Adult/ College-Aged (18–22 years old)	19	13.10%
Emerging Adulthood/Post-College Aged (22–26 years old)	1	0.69%
Adulthood (aged 26 + years old)	3	2.07%
Self-describe (Please write out)	0	0.00%
Age of Acquisition: English		
Infant (age birth - 2 years old)	72	49.66%
Toddler (age 2–4 years old)	41	28.28%
Young Childhood/Elementary School-Aged (age 4–10 years old)	26	17.93%
Pre-teen/Middle School-Aged (age 10–13 years old)	4	2.76%
Adolescent/High School-Aged (14–18 years old)	1	0.69%
Young Adult/ College-Aged (18–22 years old)	1	0.69%
Emerging Adulthood/Post-College Aged (22–26 years old)	0	0.00%
Adulthood (aged 26 + years old)	0	0.00%
Self-describe (Please write out)	0	0.00%

Means and distributions of data for DDBHH sample (*N* = 145) participants. Percentage calculated with total sample. Percentage of sample was not calculated for self-reported Race because more than one category could be selected. Education Level: Of the 47 participants who reported “NA” for Education Level, *n* = 29 of these participants can be assumed to have some undergraduate education as they were enrolled in a study recruiting from an undergraduate class. The remaining *n* = 18 individuals have an “NA” response and their education level is unknown.

### The academic anxiety inventory (AAI)

As previously mentioned, this 50-item questionnaire assessed anxiety related to five domains of anxiety: math, science, test, trait, and writing (Pizzie and Kraemer, 2019). Each domain included 10 questionnaire items related to anxiety within the academic area. Participants indicated their agreement/disagreement with each statement and responded to a 1–5 Likert scale. Some items were positively worded such that higher scores represented more positive/less anxious responses, and these were reverse scored. All 10 items in each subscale were scored and an average score was reported for each subscale for each participant (average scores fall between 1 and 5). Higher scores on each subscale indicate more negative and more anxious responses.

### Self-reported language background

For this sample of DDBHH individuals, we also had participants report their hearing status, use of assistive devices for access to sound (e.g., cochlear implants, hearing aids, etc.), age of acquisition for ASL and English, and number of years using ASL. Participants also reported their education level.

Participants rated their language use and communication strategies, including assessment of receptive and expressive communication strategies like ASL, written English, spoken English, and sign-supported language strategies such as “Signed Exact English.” For example, participants made ratings such as “Indicate your preference for using each method YOURSELF to communicate with others,” and “Indicate your preference FOR OTHERS to use each method of communication to communicate with you.” Participants made these ratings on a − 5 (Would NOT prefer to use) to +5 (Would STRONGLY prefer to use) scale, and were given a “not applicable” (NA) option. Participants were also given the opportunity to report the use of additional languages, but the analyses in this paper focused on ASL-English bilingualism.

Participants completed a short measure (six questions) assessing their comfort, skill, and identity related to expressive and receptive skills in ASL. For example, “I feel very comfortable understanding others when they use ASL to communicate with me,” and “My ability to communicate using ASL is very important to my identity.” Participants also reported other elements of language such as whether family members used ASL, and use of closed captions when growing up.

### ASL videos

Each item in the AAI and demographics section included both the English version of the item, as well as a video translation in ASL. All survey measures were administered online using the Qualtrics online survey platform (Provo, UT), which allowed the English version of each item to be paired with embedded ASL videos hosted on YouTube, and allowed the participant to control the timing of the video, rewatch it, or show a larger version of the video. Because many members of DDBHH communities report strong preferences for viewing and using ASL to communicate, these videos in the questionnaire provide information in the participants’ preferred language. These video translations were intended to be co-presented with the items in English, providing ASL-English bilingual support for the administration of the questionnaires in this study. It was not the intention of this study to provide a standalone ASL translation of the AAI that was only administered in ASL with no English provided. Thus, we did not conduct independent, blinded, forward and back translation of the ASL translations, nor did we evaluate participant responses on an ASL-only version of the questionnaire. Instead, the intention was to present all items bilingually in ASL and English simultaneously to provide access and communication for a variety of language backgrounds.

All videos were translated and signed by a female deaf signer who was fluent in English and ASL. The same deaf signer also provided ASL versions of the study instructions. Participants could choose to read the item in English, watch the video in ASL, or both. In addition, the ASL video translations for all 50 items of the AAI were evaluated against the English versions by two additional deaf individuals who were fluent in ASL and English. All translations were determined to be sufficiently equivalent across both ASL and English. Only one item was flagged as having a potentially conflicting translation, in which the item in English “I feel pleasant” was translated in ASL to have a meaning closer to “I feel friendly/open.” Overall, the items were determined to be sufficiently similar across both languages for the administration of the questionnaire.

### Procedure

Participants were recruited through flyers, emails, bulletin posts, and personal contacts and were invited to our studies first completed a screener questionnaire to determine eligibility, indicating that all participants were over the age of 18 years, could read and write in English, and had at least minimal (or better) knowledge of ASL. Participants completed an online consent form with an ASL translation available. Participants then completed the AAI as part of a randomized battery of online questionnaires. Participants also completed self-reported demographic information. According to the hypotheses of each study, participants completed additional questionnaires or tasks that were not germane to the current analyses; these analyses will be reported in additional publications of study-specific results, such as Pizzie et al., 2025. All procedures were approved by the Gallaudet University Institutional Review Board (IRB). All participants were compensated for their time in each individual study and were reimbursed with cash or a gift card.

### Analysis plan

In this study, we first wanted to evaluate the reliability of the AAI subscales in a new sample of DDBHH people. We mirrored the analysis plan that evaluated reliability in the original publication validating the AAI (Pizzie and Kraemer, 2019). Unless otherwise specified, the analyses planned in this paper represent individual and independent tests and we did not implement a multiple comparisons correction (such that Type I error rate, alpha = 0.05; García-Pérez, 2023). To evaluate reliability, we calculated the Cronbach’s *α* for each subscale, evaluating these values against a criterion of α = 0.70, as we did in the original publication of the AAI. We further evaluated the reliability of the AAI with both exploratory and confirmatory factor analyses. Using an exploratory factor analysis (principal components analysis), we evaluated the number of components, and where the eigenvalues for these components began to level off, indicating the number of components that provides an ideal solution for our data. We evaluated these results against the five-factor solution for the original AAI subscales. In addition, we calculated a confirmatory factor analysis with a five-factor solution with a varimax rotation. We then evaluated the factor loadings for each questionnaire item, giving us an understanding as to how the items aligned together along the hypothesized subscales. To compare this five-factor solution to the original structure of the original samples of the published, validated AAI, we calculated a Pearson correlation of the matrix of the factor solution with the previous factor analysis from the original publication (Pizzie and Kraemer, 2019, Table 5 undergraduate sample, *N* = 227; Note: the original sample included 236 individuals, but due to missing data values for some AAI subscales, 227 individuals were used for this analysis). This correlation will give us a measure of similarity of the current sample’s reliability with the original factor analysis solutions. Finally, we evaluated the test–retest reliability by calculating a paired *t*-test between a subset of DDBHH participants who took the AAI at two separate timepoints, and we also calculated a Pearson correlation of the scores between the timepoints, allowing us to evaluate whether these scores were sufficiently reliable across assessments. Overall, we will use these analyses to evaluate whether the five subscales of the AAI have sufficient reliability to be used within a sample of DHH adults.

In addition to evaluating the AAI, we also completed two analyses exploring unique elements of our DDBHH sample. To evaluate whether the subscales of the AAI represented independent domains of anxiety, we calculated Pearson correlations. To investigate whether DDBHH people had elevated academic anxiety, we used independent samples *t*-tests to evaluate the difference in mean scores between the originally published hearing sample (*N* = 227; Pizzie and Kraemer, 2019) and the current sample of DDBHH individuals (*N* = 145). In order to account for differences in sample size and variance, we utilized a degrees of freedom correction to Welch’s *t*-test. We utilized a Bonferroni correction to account for the five statistical tests, such that alpha = 0.01 for the *t*-tests in Research Question 3.

We used correlations to evaluate the relations between self-reported language background, and self-reported writing anxiety (AAI-Writing subscale). These questions are exploratory and we chose not to correct for multiple comparisons. We chose to focus specifically on writing anxiety because of the clear connections between writing and the domain of language. The language background variables were not normally distributed within this sample, so Spearman correlations were used to evaluate these relationships.

## Results

### Research question 1: in a DDBHH sample, is the AAI a sufficiently reliable measure?

#### RQ 1a: using Cronbach’s alpha, do all subscales have sufficient reliability?

**Figure 1:**
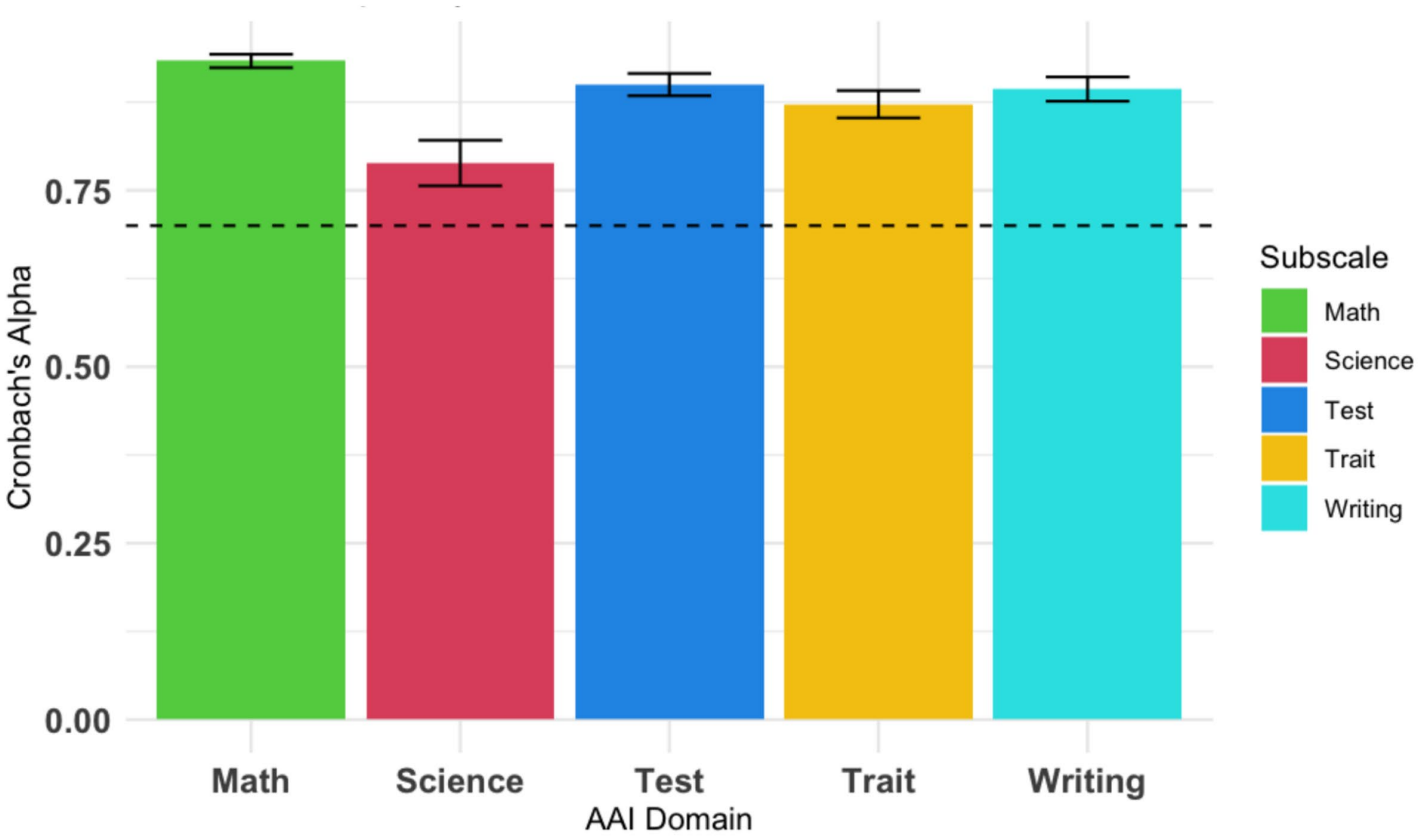
Cronbach’s α reliability measures for each AAI subscale. Cronbach’s α reliability was calculated for each AAI subscale. All subscales show reliability that is above the recommended criterion of α = 0.70, shown by a dotted line.

To evaluate reliability, we first calculated the Cronbach’s *α* for each subscale. We found that all subscales had sufficient internal reliability (Figure 1): AAI-Math: α = 0.93 (Alpha Standard Error, ASE = 0.01), AAI-Science: α = 0.79 (ASE = 0.03), AAI-Writing: α = 0.89 (ASE = 0.02), AAI-Test: α = 0.90 (ASE = 0.02), AAI-Trait: α = 0.87 (ASE = 0.02). All subscales surpassed the minimum criterion of α = 0.70, indicating that these subscales have sufficient within-subscale reliability within this DDBHH sample.

#### RQ 1b: exploring the factor structure of the AAI subscales, are hypothesized domains of math, science, writing, trait, and test anxiety represented in the data?

To determine whether the AAI data represented the hypothesized number of domains, we first conducted a principal components analysis (PCA, unrotated) using the AAI data from all 50 items for our DDBHH sample (*N* = 145). As we predicted from the five subscales, the first 5 components represented the majority of the variance in this sample, accounting for 51.3% of the variance in the sample (50 components account for 100% of the variance). The standard deviation for each of these components was above 1 (PC1: SD = 3.26, Proportion of Variance (POV) = 0.19; PC2: SD = 2.89, POV = 0.15; PC3: SD = 2.04, POV = 0.07; PC4: SD = 1.80, POV = 0.06; PC5: SD = 1.54, POV = 0.05). Additional components continued to asymptote and decreased in the proportion of variance accounted for, with each additional component accounting for 3% or less variance. This provided evidence that five components accounted for more than half of the variance within our AAI sample.

We used a maximum likelihood factor structure to confirm the hypothesized five-factor structure of the questionnaire, investigating whether the AAI items loaded onto the hypothesized factors representing math, science, writing, test, and trait subscales (Table 2). In other words, we conducted this factor analysis to investigate whether each item aligned with its hypothesized subscale. Our confirmatory five factor solution sufficiently explained the variance among these items, X^2^_(985)_ = 1409.57, *p* < 0.001, Tucker-Lewis Index of factor reliability = 0.80, RMSEA index = 0.054 [95% CI = 0.05, 0.06]. Replicating a previous analysis from the original validation of the AAI, we wanted to explore whether each AAI item would load onto a factor representing each subscale (see Table 5, Pizzie and Kraemer, 2019). A suggested factor loading of 0.3 or above is considered “significant,” but here we utilized a more stringent criterion for factor loading of 0.5 (Pizzie and Kraemer, 2019; Yong and Pearce, 2013). For all subscales, even using this more conservative criterion, the majority of items in each subscale loaded onto a factor representing that subscale: Math: 80%, Science: 60%, Writing: 100%. Test: 70%, Trait: 80% (Table 2). Using the more standard criterion of 0.3 for factor loadings, 90–100% of the items load on to the hypothesized subscale. Overall, this confirmatory factor analysis demonstrates that the items and subscales composing the AAI are also represented in our current sample of DHH individuals.

**Table 2:** Factor loadings from Maximum-Likelihood factor analysis for AAI items from DDBHH sample (*N* = 145) and hearing undergraduate sample (*N* = 227).

AAI subscale	Item	DDBHH adult sample (*N* = 145)					Hearing undergraduate sample (*N* = 227)				
		RC2	RC3	RC1	RC4	RC5	F1	F2	F5	F3	F4
Quantitative/ Science anxiety	AAI_2*	0.12	−0.26	0.22	−0.15	**0.50**	0.08	−0.26	0.27	0.19	*0.46*
	AAI_6*	0.09	0.00	0.01	0.21	*0.41*	0.11	−0.03	0.06	0.11	**0.60**
	AAI_13	*0.42*	0.14	−0.11	0.10	*0.33*	0.27	−0.02	0.04	−0.01	**0.59**
	AAI_22	−0.01	0.14	−0.06	0.28	**0.51**	0.19	0.18	0.11	0.15	**0.58**
	AAI_24*	0.11	0.18	−0.04	*0.31*	*0.42*	−0.03	0.00	0.05	0.24	*0.47*
	AAI_28	0.09	0.17	0.00	0.02	**0.67**	0.20	0.08	0.14	0.11	**0.73**
	AAI_31	0.01	−0.04	0.11	0.09	**0.61**	0.08	0.07	0.07	0.13	**0.74**
	AAI_32	0.15	0.11	0.06	0.02	**0.59**	0.09	0.20	−0.01	0.08	**0.77**
	AAI_49	0.16	0.26	−0.03	0.25	*0.41*	−0.04	0.11	−0.07	0.15	**0.70**
	AAI_50*	−0.06	−0.19	0.27	−0.10	**0.56**	0.11	−0.25	0.33	0.14	*0.37*
Math anxiety/ attitudes	AAI_7*	**0.87**	−0.07	−0.16	0.07	−0.02	**0.85**	0.06	−0.05	−0.03	0.11
	AAI_11	**0.83**	0.00	−0.07	−0.06	0.07	**0.83**	0.08	−0.02	−0.06	0.15
	AAI_12*	**0.82**	0.12	−0.08	0.09	0.03	**0.77**	0.09	0.05	0.02	0.11
	AAI_16	**0.79**	0.05	0.06	0.08	0.11	**0.71**	0.17	0.02	0.18	0.09
	AAI_26	0.27	0.04	0.00	0.16	0.09	0.24	−0.04	0.03	−0.03	*0.41*
	AAI_37*	*0.43*	−0.17	0.10	0.05	0.28	**0.51**	−0.05	0.32	0.03	*0.35*
	AAI_40*	**0.84**	0.08	−0.20	0.06	−0.04	**0.83**	0.00	−0.01	0.05	0.05
	AAI_41	**0.83**	0.15	−0.10	0.06	0.09	**0.74**	0.16	0.01	0.10	0.18
	AAI_42*	**0.81**	0.17	−0.10	0.05	−0.06	**0.64**	0.08	−0.01	0.06	0.06
	AAI_48	**0.77**	0.00	0.11	0.04	0.13	**0.72**	0.12	−0.07	0.06	0.16
Test anxiety	AAI_3*	0.18	0.20	0.31	0.11	0.07	0.05	−0.16	0.22	0.15	0.28
	AAI_10	0.07	**0.77**	0.18	0.16	0.04	0.15	**0.72**	0.02	0.28	−0.04
	AAI_19	0.12	**0.75**	0.08	0.05	−0.03	0.20	**0.70**	0.00	0.13	0.00
	AAI_21	−0.12	**0.71**	−0.05	0.10	0.08	0.00	**0.72**	0.10	0.11	0.00
	AAI_25*	0.23	*0.48*	0.08	0.22	−0.08	0.24	*0.47*	0.21	0.29	0.05
	AAI_30*	0.18	*0.40*	0.20	0.12	−0.07	0.27	*0.38*	0.16	*0.38*	0.09
	AAI_35	−0.06	**0.70**	−0.07	0.14	0.16	0.06	**0.68**	0.00	0.16	0.02
	AAI_44	0.12	**0.77**	0.05	0.10	−0.01	0.07	**0.78**	−0.08	0.15	−0.01
	AAI_46	0.07	**0.80**	−0.03	0.00	−0.01	0.03	**0.82**	−0.08	0.14	0.01
	AAI_47	0.03	**0.79**	0.05	0.06	0.12	0.13	**0.78**	0.06	0.08	0.11
Trait anxiety	AAI_1	0.07	*0.33*	0.13	*0.41*	0.03	0.02	0.23	−0.31	*0.30*	−0.09
	AAI_14	0.05	0.29	0.21	**0.51**	0.20	0.11	0.22	0.15	**0.64**	0.16
	AAI_15	0.18	*0.35*	0.06	**0.50**	0.12	0.04	0.22	−0.02	**0.63**	0.13
	AAI_17	0.13	0.00	0.04	**0.71**	0.14	0.03	0.10	0.15	**0.71**	0.06
	AAI_18*	0.07	0.00	0.08	**0.68**	−0.08	0.03	0.06	0.18	**0.70**	0.04
	AAI_20	0.03	0.09	0.09	**0.69**	0.27	0.07	*0.33*	−0.13	**0.70**	0.15
	AAI_23	−0.02	*0.35*	0.07	*0.33*	0.20	−0.07	0.17	−0.02	*0.42*	*0.32*
	AAI_29	−0.04	0.20	0.14	**0.71**	0.18	0.07	0.16	0.07	**0.74**	0.14
	AAI_33*	0.10	−0.03	0.12	**0.76**	0.10	−0.02	0.02	0.13	**0.72**	0.24
	AAI_39*	0.07	0.23	0.01	**0.50**	−0.16	0.07	0.23	0.09	**0.54**	0.11
Writing anxiety	AAI_4*	−0.08	−0.17	**0.55**	−0.08	0.09	−0.02	−0.04	**0.56**	0.05	0.00
	AAI_5	−0.12	−0.15	**0.60**	−0.13	0.30	0.02	−0.04	**0.67**	0.01	0.15
	AAI_8*	−0.04	0.09	**0.63**	0.28	−0.01	0.09	−0.08	**0.52**	*0.37*	0.08
	AAI_9	−0.05	0.27	**0.71**	0.08	−0.03	0.13	*0.35*	**0.55**	0.19	0.11
	AAI_27	0.01	0.26	**0.59**	0.03	0.08	−0.03	0.18	**0.60**	0.17	0.07
	AAI_34	−0.12	*0.33*	**0.56**	0.24	0.13	0.09	0.10	*0.43*	*0.44*	0.27
	AAI_36*	−0.02	−0.10	**0.78**	0.03	0.04	−0.12	0.12	**0.77**	−0.04	−0.01
	AAI_38*	−0.10	−0.12	**0.70**	0.09	0.03	0.00	0.02	**0.78**	0.00	−0.01
	AAI_43*	−0.03	0.09	**0.65**	0.24	−0.20	0.00	0.07	**0.72**	0.09	0.07
	AAI_45	−0.13	*0.36*	**0.62**	0.15	0.21	0.01	*0.54*	0.27	0.21	0.21

Factor loadings from maximum-likelihood factor analysis with 50 items of the AAI. Loadings on each factor representing each hypothesized subscale for math, science, writing, test, and trait anxiety. Colors are used to represent each hypothesized subscale. Factor loadings above the threshold of 0.5 are in bold, factor loadings between the threshold of 0.3 and 0.5 are in italics. Factor loadings for the DDBHH Adult sample (left) are compared to the hearing undergraduate sample in Pizzie and Kraemer (2019; *N* = 227, right). *indicates a reverse-scored questionnaire item.

Our next analysis was designed to test the alignment between the item-wise factor analysis and our previously reported results with a hearing sample (Table 2; Table 5, Pizzie and Kraemer, 2019). Replicating a previous analysis in the original validation of the AAI, we correlated the matrices of these factor results with our current DDBHH sample (*N* = 145) with the previously published sample of hearing young adults (*N* = 227; see Pizzie and Kraemer, 2019 for demographic information about this sample). We used a Pearson correlation to correlate the hypothesized overall factor structures, aligning each hypothesized factor column in the same order, such that the hypothesized factor for “math” appeared in the same column in both datasets (see Table 2). Across both the DDBHH and hearing samples, the factor structures were highly correlated with one another, *r*(248) = 0.78, *p* < 0.001, 95% CI = [0.725, 0.824], *r^2^* = 0.61. Although the magnitude of this correlation is significantly less than the correlation reported between the undergraduate sample and sample of high schoolers in the original publication (*r*(248) = 0.86, *p* < 0.001, *r^2^* = 0.74, comparing correlation size: *Z* = 3.28, *p* < 0.001), it is clear that the factor structures are still highly and significantly correlated between the DDBHH and hearing samples. We also correlated the factors within each domain between the DDBHH and hearing factor analyses, calculating the Pearson correlation between the columns matching the subscale domains and found high correlations between domains: Math: *r* = 0.77 (*r^2^* = 0.59), Science: *r* = 0.80 (*r^2^* = 0.64), Writing, *r* = 0.82 (*r^2^* = 0.67), Test: *r* = 0.83 (*r^2^* = 0.69), Trait: *r* = 0.78 (*r^2^* = 0.61).

#### RQ 1c. Does the AAI have sufficient test-retest reliability in a DDBHH sample?

We wanted to explore whether each of the subscales in the AAI had reasonable test–retest reliability. In a subset of our participants (*n* = 45), participants completed the AAI at least twice by participating in multiple studies run by the lab, and the time between assessments ranged between 13 and 762 days with an average of 325.24 days (SD = 239.85) between administrations of the survey. We computed a paired *t*-test to compare scores each subscale to determine whether scores significantly changed from one administration to the next and used Pearson correlations to assess the extent to which these scores were related.

Math scores on the AAI-Math subscale were highly correlated, *r*(43) = 0.94, *p* < 0.001, *r^2^* = 0.88. These scores did not significantly change across timepoints, *t*(44) = 1.58, *p* = 0.12 (Mean difference = 0.12). Scores on the AAI-Science subscale were also highly correlated across timepoints, *r*(43) = 0.97, *p* < 0.001, *r^2^* = 0.94, and did not significantly change between timepoints, *t*(44) = 1.30, *p* = 0.20 (Mean difference = 0.07). Scores on the AAI-Test subscale were highly correlated, *r*(43) = 0.91, *p* < 0.001, *r^2^* = 0.82, and these scores did not significantly change between timepoints, *t*(44) = 0.87, *p* = 0.40 (Mean difference = 0.07). Scores on the AAI-Trait subscale were highly correlated across timepoints, *r*(43) = 0.92, *p* < 0.001, *r^2^* = 0.84, and did not significantly change between timepoints, *t*(44) = −0.55, *p* = 0.59 (Mean difference = −0.04). Finally, scores on the AAI-Writing subscale were also highly correlated across timepoints, *r*(43) = 0.97, *p* < 0.001, *r^2^* = 0.94, and did not significantly change between timepoints, *t*(44) = 1.16, *p* = 0.25 (Mean difference = 0.06). All subscales were highly correlated with one another, and did not significantly differ between timepoints, indicating high test–retest reliability.

#### RQ 2. Do all the subscales represent related but independent factors?

In this analysis, we wanted to explore intercorrelation between the subscales of the AAI. In the creation of the original measure (Pizzie and Kraemer, 2019), questions for the subscales were selected such that the correlations between subscales were reduced, indicating that the subscales represented more separable domains of anxiety compared to previous measures that showed a high degree of intercorrelation (i.e., *r* = 0.6–0.75). We utilized Pearson correlations to evaluate the relations between each of the subscales, and results are presented in Table 3.

**Table 3:** Pearson correlations between AAI domains of anxiety from DDBHH (*N* = 145) and Hearing (*N* = 227) samples.

AAI Domain	Math	Science	Test	Trait	Writing
Math	--	0.31**	0.25**	0.15*	0.08
Science	0.28**	--	0.19**	0.38**	0.31**
Test	0.17*	0.21*	--	0.49**	0.28**
Trait	0.18*	0.37***	0.43***	--	0.34**
Writing	−0.13	0.19*	0.23**	0.31***	--

Pearson correlation coefficients for correlations between each AAI subscale. *p < 0.05, **p < 0.01, ***p < 0.001. Data presented below the diagonal is from the present sample of DDBHH individuals (*N* = 145) and data above the diagonal are presented from the original undergraduate sample (Pizzie and Kraemer, 2019; Table 6, *N* = 227 undergraduate students).

As demonstrated in Table 3, the subscales have relatively low intercorrelation between the scales, such that all correlations are below a magnitude of *r* = 0.5. Comparing the correlations from our current study of DDBHH participants (*N* = 145) to the previously published undergraduate sample (*N* = 227, Table 6; Pizzie and Kraemer, 2019), we observe a similar pattern of intercorrelation between the subscales in our current sample and a sample of hearing undergraduate students. Results from our current sample (below the diagonal) and the original sample (Pizzie and Kraemer, 2019 from Table 6 above the diagonal) are presented in Table 3; the correlations are comparable across both samples. In RQs 1–2, we established that the AAI had high reliability, a similar factor structure, and a high test–retest reliability in a novel sample of DDBHH people, showing very similar results to the original psychometric data in hearing participants.

#### RQ 3: Do DDBHH individuals report increased academic anxiety compared to a hearing sample?

In RQ 3 we sought to add to the literature through more exploratory analyses, investigating whether our DDBHH sample (*N* = 145) reported elevated anxiety across any of the subscales compared to a hearing sample (*N* = 227). The undergraduate sample was drawn from the original data published in Pizzie and Kraemer (2019), and was also compared in the factor analysis (RQ 1b).

We conducted independent t-tests comparing mean subscale scores between the DDBHH and hearing samples; results are shown in Table 4. Our results suggest that the DDBHH sample reported increased science anxiety and trait anxiety compared to the hearing sample. The DDBHH sample also reported slightly elevated math anxiety compared to the hearing sample, although this comparison had a *p*-value of 0.048, which was not considered to be statistically significant with an alpha level that was corrected for multiple comparisons. Comparisons of self-reported test anxiety and writing anxiety were not significantly different between groups.

**Table 4:** Comparing mean scores on each AAI subscale between DDBHH (*N* = 145) and Hearing (*N* = 227) samples.

AAI subscale	*df*	*t*	*p*	95% CI mean difference	Cohen’s *d*	Group difference
Math	275.73	−1.99	0.048	−0.35–−0.002	0.21	*ns*
Science	324.28	−5.48	<0.001***	−0.43–−0.20	0.58	Hearing < DDBHH
Test	310.18	0.29	0.77	−0.12–0.16	0.03	*ns*
Trait	309.97	−3.02	0.003**	−0.37–−0.08	0.32	Hearing < DDBHH
Writing	284.08	0.53	0.60	−0.11–0.18	0.06	*ns*

Independent sample t-tests comparing DDBHH and Hearing samples on mean levels of each domain of academic anxiety. In these analyses, we have utilized a Bonferroni correction to compensate for five statistical tests related to this research question, α = 0.01, ** < 0.01, *** < 0.001. Degrees of Freedom (df) were corrected to account for the imbalance in sample sizes. 95% Confidence Interval (CI) of the mean difference between each group is reported. *ns* indicates “not significant” or no differences between samples.

#### RQ 4: How does writing anxiety relate to language preferences in a DDBHH sample?

In RQ 4, we explored how a particular domain of anxiety, writing anxiety, was associated with self-reported language background in a DDBHH sample.

First, we evaluated whether participants’ self-reported preference for written English was associated with writing anxiety (AAI-Writing subscale). Self-reported preferences for written English as an expressive communication strategy were negatively associated with decreased AAI-Writing scores, *r_s_*(141) = −0.23, *p* = 0.007, *r^2^* = 0.05. The more that participants reported an increased preference for written English as a communication strategy, the less they reported experiencing writing anxiety. Self-reported preferences for receptive written English (reading what others have written) was not significantly related to AAI-Writing scores, *r_s_*(141) = −0.13, *p* > 0.05.

We also explored whether writing anxiety was associated with any other communication strategies, including spoken English, ASL and SEE, and assessed preferences across both receptive and expressive communication strategies. Neither expressive nor receptive spoken English preference was significantly associated with writing anxiety, all *p*’s > 0.05. Neither expressive nor receptive ASL preference was significantly correlated with self-reported writing anxiety, *p*’s > 0.05. Similarly, neither expressive nor receptive SEE preference was significantly associated with writing anxiety, *p*’s > 0.05. Overall, we find that self-reported preference for using written English as a communication strategy was inversely correlated with writing anxiety, and other communication strategies were not related to self-reported language anxiety. Additional correlations between other subscales of the AAI and self-reported preferences for communication strategies are reported in Supplementary Table 1.

We also explored an additional measure of self-reported comfort with ASL, which provided more elaborated information beyond self-reported communication preferences. We investigated whether self-reported comfort with ASL was associated with self-reported writing anxiety. We find that increased comfort with ASL was negatively associated with writing anxiety, *r_s_*(142) = −0.17, *p* = 0.048, *r^2^* = 0.03, such that increased comfort using ASL was associated with decreased reports of writing anxiety.

## Discussion

In this study, we aimed to establish a bilingual administration of the Academic Anxiety Inventory (AAI) as an appropriate and reliable measure of five different kinds of academic anxiety in d/Deaf, DeafBlind, and hard of hearing (DDBHH) people. Our results showed that the AAI had sufficient reliability within each subscale (RQ 1a), and we found that the overall factor structure was well-represented by our hypothesized subscales (RQ 1b). In investigating the reliability of the AAI, we also found that the test–retest reliability of each subscale of the AAI was sufficiently high (RQ 1c). Similar to the validation of the original AAI questionnaire (Pizzie and Kraemer, 2019), we found that the AAI subscales represented independent domains of anxiety associated with math, science, test, trait, and writing anxieties, reducing the amount of intercorrelation between the domains of anxiety (RQ 2). Finally, we wanted to explore two areas that were more specifically related to our unique sample of DDBHH individuals: our results showed that DDBHH individuals experience greater academic anxiety related to science and trait (general) anxiety compared to a hearing sample (RQ 3). We also found that writing anxiety was inversely associated with communication preferences for written English, and inversely related to comfort with ASL (RQ 4). Across these research questions and analyses, our results suggest that the bilingual administration of AAI is a reliable measure that can be used with DDBHH participants to identify five domains of self-reported academic anxiety.

Although much of the results of this study confirmed similar results to the original psychometric analysis, it is also important to consider the variability in results, as well. In this study, factor structure was highly correlated to the factor structure in the original validation analyses (RQ 1b; Pizzie and Kraemer, 2019). However, this correlation between factor structures was lower than the intercorrelation reported in the original paper between a hearing adult sample and adolescent sample. Although the correlation between factor structures of the DDBHH and original validation sample of hearing adults is still very high, there are some important considerations related to our DDBHH sample that may have slightly decreased this correlation. Our sample size (*N* = 145) is smaller than the original validation sample and may be more heterogeneous with regard to language and educational backgrounds. Moreover, DDBHH individuals were responding to a bilingual version of the AAI instead of a monolingual English version, which may have slightly altered response patterns. Interestingly, one way in which responses may have differed between samples is that we observe more consistent factor loadings along the writing anxiety subscale in the DDBHH sample (two items were below the 0.5 threshold in the original validation sample). All these factors may have contributed to slightly lower correlation between the DDBHH sample and the original validation sample, though these patterns of responses are still highly correlated.

Further, conducting research with a sample of DDBHH individuals allowed us to explore some questions that would be more unique to the characteristics of DDBHH communities. For example, because some members of DDBHH communities may experience increased barriers in educational environments (Garberoglio et al., 2019; Solomon, 2021), we hypothesized that DDBHH individuals would self-report increased academic anxiety, especially for STEM or language-based domains. Specifically, we hypothesized our sample would report increased math, science, writing, and general (trait) anxieties. Instead, our results only partially confirmed this hypothesis, such that compared to a hearing sample of undergraduate students, DDBHH individuals reported increased science anxiety and trait (general) anxiety (RQ 3). The increased anxiety about science is consistent with our hypothesis. We expected that because individuals with disabilities are underrepresented in STEM (Solomon, 2021), and many DDBHH individuals have faced additional challenges in STEM achievement due to lack of accessibility. Past research (Kushalnagar et al., 2019; Kushalnagar et al., 2020a) also suggests that DDBHH people may be at risk for experiencing heightened generalized anxiety. Our results were aligned with this previous work suggesting that our DDBHH sample reported increased trait anxiety. We did not find any evidence that DDBHH individuals reported elevated test anxiety or writing anxiety compared to our hearing sample of participants.

This paper also investigated novel relations between writing anxiety, a facet of language anxiety (Kim et al., 2025), and self-reported language preferences in our DDBHH sample (RQ 4). Because DDBHH communities have diverse language backgrounds and preferences, we investigated how (English) writing anxiety was associated with self-reported language preferences across ASL and English. These analyses are exploratory, and further research should continue to follow up on these results. Our results show that positive preferences for using written English as an expressive communication strategy were associated with less self-reported writing anxiety, providing some convergent validity for the construct. Interestingly, self-reported ASL comfort was also inversely correlated with writing anxiety, such that if a participant reported more comfort with ASL, they reported less writing anxiety. Of course, the reverse may also be true, that those with lower writing anxiety may be more likely to choose ASL or written English as a communication strategy. That our results show that increased comfort with ASL is associated with benefits for anxiety about written language suggests an advantage for signed language bilingualism in DDBHH individuals. This may serve as an example of cross-linguistic transfer that supports bilingual competence, such that skills and patterns of thought in a dominant language may also transfer to a secondary language (Odlin, 2005; Zhang et al., 2024). Building skills in an accessible signed language has been shown to benefit broader language skills (Finton et al., 2024; Humphries et al., 2012, 2014; Pontecorvo et al., 2023), and our results further suggest that more ASL comfort may be related to building balanced bilingual skills by reducing writing anxiety.

## Limitations and future directions

These results have some important limitations that must be considered. First, our DDBHH participants reported a high level of educational attainment, potentially limiting the generalizability of these results to other DDBHH people. The majority of our sample reported having completed at least some undergraduate education, and approximately 20% reported having an advanced degree, such as a Ph. D. or similar degree. Although participants were not recruited on the basis of education level, many of the participants were recruited from a university campus and surrounding area, resulting in a sample that has high educational attainment. This high level of education may not be representative of larger DDBHH communities, who often have decreased educational achievement, with significantly fewer undergraduate students earning a bachelor’s degree, and more students enrolling in certificate programs (Bloom and Palmer, 2023; Garberoglio et al., 2019; Solomon, 2021) as compared to their hearing peers. If anything, we believe that this may have decreased the amount of academic anxiety reported by our sample. Increased educational attainment may be associated with developing coping strategies that could reduce levels of anxiety.

Although we do observe that some domains of anxiety showed elevated scores in our DDBHH sample compared to a hearing undergraduate sample, it is possible that our sample may underestimate the overall levels of anxieties in DDBHH communities and their impact on educational outcomes. Additional research with a more diverse sample of DDBHH individuals who have lower levels of educational attainment may be more representative of DDBBHH communities. For example, inclusion of more DDBHH people who did not finish high school, who received a high school diploma but did not attend college, or who attended junior college or community college might provide additional insights into how academic anxiety affects DDBHH individuals who did not attend a 4-year bachelor’s degree-granting institution. Our current analyses did not compare across education levels, but future research could explore whether individuals with lower educational attainment experience higher levels of academic anxiety. If individuals experience high levels of academic anxieties, future research should also explore the relationship to educational attrition in DDBHH communities. Much of the research on academic anxiety, including the original validation of the original AAI, has included highly educated samples with students who attend universities with competitive admissions (Pizzie and Kraemer, 2019). Further development and validation analyses of the AAI and other explorations of academic anxieties would benefit from recruiting samples of participants with lower educational attainment to understand how increased anxiety may create barriers for educational success.

An additional limitation of our current administration of the AAI is that the translated items in ASL were not fully validated as a separate translation, and we did not utilize a separate forward- and back-translation of the AAI items in ASL. We recognize that this potentially limits the interpretation of our current results, as our analyses pertain only to the reliability and validity of a bilingual administration of the AAI, not to a validated ASL-only version. This kind of translation protocol would have been more appropriate if our goal was to establish and validate a separate administration of the AAI that was administered only in ASL. For researchers who might want to administer an ASL-only version of the questionnaire for DDBHH individuals who would prefer or rely on ASL as their primary language, we would encourage these researchers to take the appropriate steps for validating a “standalone” ASL version of the questionnaire before interpreting the results. However, a fully ASL version of the questionnaire might not be accessible to all DDBHH individuals. For example, a fully-ASL version of the questionnaire might not be accessible to a hard of hearing individual who never learned ASL. Our intention was to utilize a bilingual administration of this questionnaire for accessibility to DDBHH individuals who use the ASL translations to view the questionnaire. For each questionnaire item, each individual could flexibly read the English, watch the ASL video, or both. As a result, all items were presented in both ASL and English, and we utilized a separate analysis to ensure that the English and ASL items were sufficiently similar so as not to create any confusion or discrepancy between languages. Because of the setup of the questionnaire administration, we could not observe whether the participants preferred the English or ASL versions of the questions. After consulting with DDBHH collaborators and research team members, presenting both English and ASL items simultaneously was determined to be the best, most accessible approach for a wide range of linguistic backgrounds and preferences in DDBHH communities. Future research could compare administrations of English-only, bilingual, and ASL-only administrations of the AAI to determine whether the language of questionnaire administration influences self-reported level of anxiety. This might elucidate how language preferences and fluency are related to reporting of anxiety levels. Our intention is to continue to prioritize accessible and culturally-appropriate measures that consider the diversity of language and educational backgrounds of DDBHH communities.

Future directions for this research should also explore how specific characteristics or identities within the deaf community are associated with different profiles of academic anxieties. For example, exploring differences in academic anxieties between hearing status, use of assistive devices, and language background profiles would provide additional insight into DDBHH communities and their experiences with education and academic environments. Although the present study focused on DDBHH individuals, including and comparing perspectives between deaf identities, or expanding to focus on Deaf Disabled people, Deaf Autistic people, people with auditory processing disorders, or other deaf identities would provide valuable insight into experiences of academic anxiety. Exploring differences in language experiences, such as proficiency, age of language acquisition, and other factors related to bilingual language acquisition in DDBHH communities should be explored in relation to language anxieties, such as anxiety about English writing, reading, and signed language anxiety (Kim et al., 2025).

Future directions should also explore the associations between academic anxieties in DDBHH individuals, socioeconomic status, and educational accessibility. Past research has suggested that socioeconomic status may play an important role in math anxiety (Rozek et al., 2019), and underrepresented individuals may be more likely to experience academic anxieties. Lack of accessibility in educational environments is a pervasive problem for many DDBHH students (Solomon, 2021), and lack of access or accommodations, or lack of resources from low socioeconomic status, may contribute to increased academic anxieties. Although the current study did not explore socioeconomic status or educational accessibility, investigating these factors, and their relation to academic anxieties and academic achievement would be valuable future directions for this research. Longitudinal design and more advanced statistical analyses would also provide stronger evidence for the relations suggested here between anxiety and performance.

## Conclusion

Overall, this study provided important evidence that the AAI may be a reliable and appropriate tool for DDBHH communities, although further studies with more diverse samples are needed. The AAI has potential practical applications with DDBHH communities, such as early identification of high academic anxieties, which have the potential to detract from academic performance in a specific domain. Further, this questionnaire has potential to be used in designing accessible interventions for DDBHH students in counseling, psychological programs, or programs focused on supporting language development or participation in STEM. The bilingual version of the AAI has potential for a wide variety of applications with DDBHH individuals, demonstrating the utility of this questionnaire beyond the domain of research. The bilingual version of the AAI used for this study presented items in both ASL and written English, making the questionnaire more accessible and culturally appropriate for members of DDBHH communities. Academic anxieties can create obstacles for learning, and if our goal is to create better and more accessible learning environments that support a variety of diverse students, we must address anxiety in educational environments for all students to thrive.

## Data Availability

Due to the small nature of the DDBHH communities, the researchers are not able to publicly share all behavioral data collected to protect participant confidentiality and privacy, as multiple pieces of demographic information can be used to triangulate individual participants’ data. Only deidentified compiled data with demographic information removed will be available for sharing through the Open Science Framework. Supplementary materials, an appendix of materials, and preprint versions of this manuscript are available through the Open Science Framework through PsyArXiv: https://osf.io/j3wfz/files/osfstorage Researchers should contact the corresponding author to request access to raw data or data that includes demographic information.
