# Supplementary material for: Assessing academic anxiety in d/Deaf, DeafBlind, and hard of hearing individuals
Source: Front Psychol. 2026 Jan 29;17:1663756. doi: 10.3389/fpsyg.2026.1663756 (PMC12894224; doi:10.3389/fpsyg.2026.1663756)
Supplement: Supplementary file 1 [file Data_Sheet_1.pdf]

Supplementary Materials

Supplementary Results

Supplementary Table 1. Correlation table between AAI Subscales and Communication Strategies

**Language Preference**	Range								
**Expressive**	Min	Max	Mean	NA	AAI-Science	AAI-Math	AAI-Test	AAI-Trait	AAI-Writing
ASL	-3	5	4.22	1	-.13	-.005	.01	-.06	-.06
Spoken English	-5	5	0.28	17	-.06	.06	.12	.17	.02
Written English	-5	5	3.21	2	-.07	.107	.08	.06	**-.23**
SEE	-5	5	-1.86	22	**.20**	-.009	.17	.07	-.06
Other	-5	5	0.84	112					
**Receptive**									
ASL	-4	5	4.26	1	**-.20**	-.04	.05	.02	-.10
Spoken English	-5	5	-0.38	3	.09	-.03	.14	**.22**	.15
Written English	-5	5	3.41	1	-.09	.09	.04	.02	-.14
SEE	-5	5	-1.14	20	.09	-.11	.09	.03	-.08
Other	-5	5	0.43	116					

Note. Table of descriptive stats and Spearman correlations between expressive and receptive communication strategies and subscales of the AAI. Correlations in bold are significant at the α = .05 level.

Supplementary Figure 1. Distribution of Mean AAI Subscale Scores for d/Deaf and Hard of Hearing Samples.

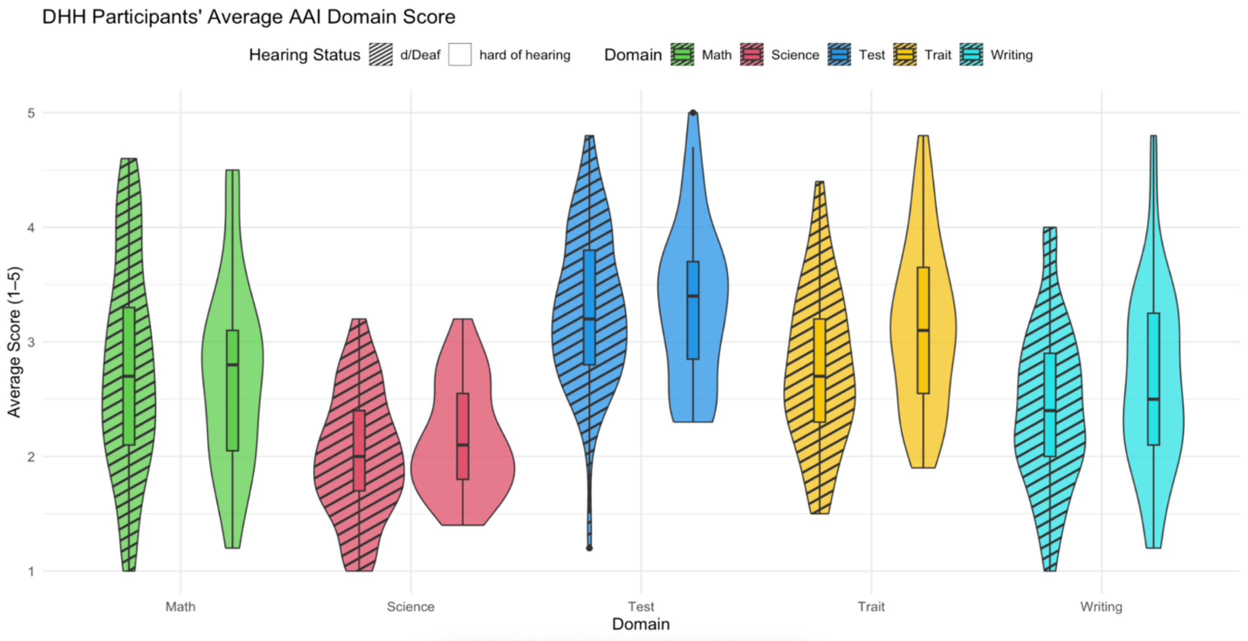

Note. Mean scores and distributions for each AAI subscale: Math, Science, Test, Trait, and Writing, comparing d/Deaf (*n* = 101) and Hard of Hearing (*n* = 39) participants. Here we did not use significance testing to compare differences between groups because of the largely unequal sample sizes.
